# Concentración del edta en la efectividad de la terapia fotodinámica antimicrobiana con curcumina sobre biofilm de *streptococcus mutans*

**DOI:** 10.21142/2523-2754-1401-2026-277

**Published:** 2025-12-28

**Authors:** Stephanie Esther Silva Urco, Sayuri Yasira Miguel Soto, Raúl Mauricio ¡ Olaechea Alejo, Gabriel Nima

**Affiliations:** 1 Carrera de Estomatología, Universidad Científica del Sur. Lima, Perú. stephaniesilvaurco@gmail.com sayurimiguelsoto@gmail.com gabrieln_b@yahoo.com Universidad Científica del Sur Carrera de Estomatología Universidad Científica del Sur Lima Peru stephaniesilvaurco@gmail.com sayurimiguelsoto@gmail.com gabrieln_b@yahoo.com; 2 Laboratorio Húmedo Experimental, Universidad Científica del Sur. Lima, Perú. ramaolal2@gmail.com Universidad Científica del Sur Laboratorio Húmedo Experimental Universidad Científica del Sur Lima Peru ramaolal2@gmail.com

**Keywords:** Streptococcus mutans, terapia fotodinámica antimicrobiana, ácido etilendiaminotetracético, curcumina, Streptococcus mutans, antimicrobial photodynamic therapy, ethylenediaminetetracetic acid, curcumin

## Abstract

**Objetivo:**

Determinar la concentración óptima de ácido etilendiaminotetracético (EDTA) en la terapia fotodinámica antimicrobiana (aPDT) mediada por curcumina y luz LED azul sobre biofilms de *S. mutans*.

**Materiales y métodos:**

Se utilizó biofilms de *S. mutans* cultivados sobre discos de resina y expuestos a dos concentraciones de EDTA (1% y 17%). Los grupos experimentales incluyeron: control negativo, control positivo (clorhexidina), EDTA al 1%, EDTA al 17%, curcumina 100 μM, curcumina 100 μM + EDTA al 1%, y curcumina 100 μM + EDTA al 17%, con y sin exposición a luz LED azul, excepto el control positivo. La viabilidad bacteriana se obtuvo con el recuento de unidades formadoras de colonias (UFC/ml) después de la aplicación de los tratamientos. Los datos fueron evaluados mediante un ANOVA de un factor ajustado por un modelo lineal generalizado (GLM) (p < 0,05).

**Resultados:**

Los grupos tratados con curcumina y curcumina + EDTA al 1%, activados con luz LED azul, mostraron la mayor reducción en la viabilidad bacteriana. La combinación con EDTA al 17% no mejoró significativamente el efecto de la aPDT.

**Conclusiones:**

La adición de EDTA no potenció significativamente la actividad de la aPDT mediada por curcumina frente a biofilms de *S. mutans*. La concentración del 1% de EDTA mostró resultados comparables al uso de curcumina sola, lo cual sugiere que concentraciones más altas no aportan un beneficio adicional.

## Abstract (en)

**Objective:**

Evaluate the optimal concentration of ethylenediaminetetracetic acid (EDTA) in curcumin-mediated antibacterial photodynamic therapy (aPDT) with blue LED light on *S. mutans* biofilms.

**Materials and Methods:**

Biofilms of *S. mutans* grown on resin discs were used, exposed to two concentrations of EDTA (1% and 17%). The experimental groups included: negative control, positive control (chlorhexidine), 1% EDTA, 17% EDTA, 100 μM curcumin, 100 μM curcumin + 1% EDTA, and 100 μM curcumin + 17% EDTA, with and without exposure to blue LED light, except for the positive control. Bacterial viability was assessed by counting colony-forming units (CFU/ml) after treatment application. Data were analyzed using one-way ANOVA adjusted by a generalized linear model (GLM) (p < 0,05).

**Results:**

The groups treated with 100µM curcumin and 100 μM curcumin + 1% EDTA, activated with blue LED light, showed the greatest reduction in bacterial viability. The combination with 17% EDTA did not significantly enhance the effect of aPDT.

**Conclusions:**

The addition of EDTA did not significantly enhance the activity of curcumin-mediated aPDT against *S. mutans* biofilms. The 1% EDTA concentration showed results comparable to the use of curcumin alone, suggesting that higher concentrations do not provide additional benefit.

## INTRODUCCIÓN

La cavidad oral alberga una amplia diversidad microbiana que forma comunidades organizadas denominadas biofilms. Algunas de estas comunidades están implicadas en la etiología de enfermedades orales como la caries dental, las infecciones pulpares y las enfermedades periodontales ^1^. Dentro de este ecosistema, *Streptococcus mutans* (*S. mutans*) ha sido identificado como uno de los principales agentes etiológicos de la caries dental, debido a su capacidad de sintetizar polisacáridos extracelulares como glucanos y fructanos, mediante las enzimas glucosiltransferasa y fructosiltransferasa ^2^^,^^3^.

Actualmente, el tratamiento de la caries dental implica la remoción mecánica de la dentina infectada seguida por su restauración con resinas compuestas ^4^. En cuanto a las estrategias preventivas, se utilizan el barniz de flúor, los selladores dentales y soluciones antisépticas como la clorhexidina. Aunque la clorhexidina al 0,2% presenta buena actividad frente a bacterias grampositivas, su eficacia disminuye contra gramnegativas y su uso prolongado puede inducir efectos adversos como disbiosis oral, disminución del pH salival, aumento del riesgo de caries y resistencia bacteriana ^5^^-^^8^.

Frente a estas limitaciones, la terapia fotodinámica antimicrobiana (aPDT) ha emergido como una alternativa prometedora. Esta técnica se basa en la activación de un fotosensibilizador por una fuente de luz en presencia de oxígeno, lo que genera especies reactivas de oxígeno (ROS) capaces de inducir daño celular irreversible en microorganismos ^9^^,^^10^. Tradicionalmente, la aPDT en odontología ha empleado láseres rojos (630-700 nm) y fotosensibilizadores como el azul de metileno o azul de toluidina ^11^^-^^13^. No obstante, la necesidad de adquirir una fuente láser ha limitado su uso clínico rutinario ^14^^-^^17^.

Varios autores, han propuesto el uso de fuentes de luz LED azul, comúnmente presentes en las unidades de fotopolimerización odontológicas, como una estrategia para superar esta barrera ^18^^,^^19^. Sin embargo, el empleo de estas unidades requiere un fotosensibilizador con afinidad espectral adecuada. En este contexto, la curcumina, un compuesto natural con propiedades antimicrobianas y un espectro de absorción de 300-500 nm, se presenta como una opción viable ^20^^-^^23^.

Paralelamente, el ácido etilendiaminotetraacético (EDTA), ampliamente utilizado como irrigante en endodoncia por su capacidad quelante, ha sido sugerido como coadyuvante para potenciar la eficacia antimicrobiana de la aPDT ^24^^,^^25^. Estudios recientes han reportado que concentraciones bajas de EDTA (0,4%) combinadas con curcumina mejoran la actividad antibacteriana frente a patógenos orales, pero hasta el momento no se ha establecido cuál es la concentración óptima de EDTA que maximice este efecto ^20^^,^^23^. Por lo tanto, el objetivo del presente estudio fue determinar la concentración más efectiva de EDTA en la terapia fotodinámica antimicrobiana con curcumina y luz LED azul sobre biofilm de *S. mutans*.

## MATERIALES Y MÉTODOS

### Diseño del estudio

Se realizó un estudio experimental *in vitro*, en el Laboratorio Húmedo Experimental de la Universidad Científica del Sur, este estudio fue aprobado por la Facultad de Odontología (PRE-8-2024-00052) y recibió la exoneración del comité de ética. El diseño se elaboró siguiendo los lineamientos de la guía CRIS. 

### Cepa bacteriana y condiciones de cultivo

En este estudio se utilizó la cepa *S. mutans* ATCC 25175, conservada a −70 °C en glicerol al 20% (v/v). Una alícuota fue sembrada en caldo BHI y cultivada durante 16 h a 37 °C en microanaerobiosis para su activación.

### Preparación de fotosensibilizantes

La curcumina (C1386-5G, Sigma-Aldrich, S. Louis, MO, EE. UU.) se disolvió en etanol al 99% para preparar una solución madre de 10 mM y otra de EDTA al 17% (PREVEST DenPro Limited, Jammu, India). El pH se ajustó de ambas soluciones a 7,0 utilizando un pH-metro multiparámetro (Hach Company, Loveland, CO, EE. UU.). Las alícuotas de curcumina (2 ml) fueron almacenadas en tubos ámbar a −20 °C hasta su uso.

### Fuente de luz 

La unidad LED azul (Elipar 3M Deep Cure, Solventum, St. Paul, MN, EE. UU.), que presentó una irradiancia de 1725 mW/cm², con un espectro concentrado entre 425 y 515 nm y un pico de emisión alrededor de 448 nm y punta de 10 mm de diámetro. La activación para todas las muestras se realizó a 1 mm de distancia, en dos ciclos de 20 s, con un intervalo de 5 s entre ellos. 

### Discos para el crecimiento del biofilm

Los discos de resina (7 mm diámetro × 0,8 mm altura) fueron fabricados en una impresora 3D (Sonic Mini 8K, Phrozen Technology, Hsinchu City, Taiwán). El programa Matter Control 2.0 fue utilizado para generar un modelo digital de los discos, el cual fue exportado a un formato STL (*Standard Tessellation Language*) utilizando el programa Chitubox (Chitu Systems; Guangdong, China), donde se agregaron los soportes y se realizó la segmentación para la impresora. La impresión se realizó con una resolución de 0,01 mm y utilizando una resina en base PLA (eSun, Shenzhen, China) de color blanco. Los discos se fotocuraron durante 10 min tras el lavado con alcohol isopropílico al 99%. Finalmente, los discos fueron esterilizados mediante exposición a luz UV-B (longitud de onda de 280-310 nm) durante 10 min por lado y almacenados en placas de 24 pozos. 

### Formación del biofilm

Para el crecimiento del biofilm, un inóculo de *S. mutans* se preparó a partir de una muestra de 10 µl del *stock* de *S. mutans*, que fue cultivado *overnight* en condiciones de microanaerobiosis (16 h, 37 °C) en medio BHI. El inóculo bacteriano fue ajustado a una concentración final de 1 × 10⁵ UFC/ml (0,5 de la escala de McFarland) en medio BHI suplementado con sacarosa al 1%. Los discos fueron incubados en este medio (24 h, 37 °C, microanaerobiosis) para permitir la formación del biofilm sobre su superficie, colocando los discos en una placa de 24 pozos. 

### Grupos experimentales y aplicación de tratamientos

Los 13 grupos experimentales (n = 4) fueron establecidos, incluyendo controles negativos, positivos (clorhexidina 2%), EDTA (1% y 17%), curcumina (100 µM) y combinaciones curcumina + EDTA. Todos los grupos fueron evaluados con y sin fotoactivación, excepto el control positivo (Tabla 1). Se realizaron tres repeticiones experimentales.

**Tabla 1:** Media ± Desviación estándar (Log UFC /mL) de los grupos experimentales

	Fotoactivación	
	Sin fotoactivación	Con fotoactivación
Control	5,94 ± 0,16 Ac*	6,07 ± 0,24 Abc*
EDTA 1%	6,19 ± 0,05 Abc*	6,35 ± 0,21 Aab*
EDTA 17%	6,64 ± 0,30 Aa*	6,73 ± 0,30 Aa*
Curcumina 100uM	5,97 ± 0,35 Ac*	5,55 ± 0,63 Bd*
Curcumina 100uM + EDTA1%	6,54 ± 0,35 Aab*	5,63 ± 0,37 Bd*
Curcumina 100uM + EDTA17%	6,75 ± 0,32 Aa*	5,76 ± 0,48 Bcd*
Clorhexidina	0,00 ± 0,00	

Letras iguales indican que no existen diferencias estadísticamente significativas (letras mayúsculas comparan grupos con y sin fotoactivación, letras minúsculas comparan grupos para una misma condición de aplicación de luz) en comparaciones según ANOVA de dos factores y corrección de Bonferroni (p < 0,005) con modelo GLM. El asterisco indica diferencias estadísticas con el control según test de ANOVA de un factor y post hoc de Dunnet (p < 0,005) con modelo GLM.

Posterior a la formación del biofilm, los discos fueron lavados dos veces con solución salina y tratados según el grupo asignado. En los grupos con aPDT, se realizó una preirradiación de 1 min con el fotosensibilizador, seguida por una irradiación (2 × 20 s, con intervalo de 5 s).

### Conteo de unidades formadoras de colonias

Tras los tratamientos, los discos fueron colocados en microtubos con 1 ml de solución salina y sometidos a sonicación durante 1 min para desprender el biofilm de la superficie de los discos. Se realizaron diluciones seriadas (hasta 10^-5^) de todas las muestras, 3 gotas (10 µL) de cada una de las diluciones fueron sembradas por triplicado en agar BHI. Las placas fueron incubadas (48 h, 37 °C, microanaerobiosis) y se contaron las unidades formadoras de colonias (UFC/ml) con la ayuda de un estereoscopio. Se realizaron tres experimentos independientes.

### Análisis estadístico

Los datos de UFC/ml fueron transformados a logaritmos (Log UFC /ml). Las pruebas de Shapiro-Wilk y Levene se aplicaron para evaluar normalidad y homogeneidad. Ante la violación de estos supuestos (p < 0,05), se utilizó un modelo lineal generalizado (GLM) para el análisis de dos factores (tratamiento * luz), seguido de comparaciones por pares de medias marginales estimadas con corrección de Bonferroni. Además, las diferencias entre cada grupo y el control positivo se evaluaron mediante un ANOVA de un factor (tratamiento), ajustado por un modelo lineal generalizado (GLM), seguido por comparaciones *post hoc* de medias marginales estimadas con corrección de Bonferroni. El nivel de significancia se fijó en 0,05. Todos los análisis se realizaron en SPSS v30 para macOS.

## RESULTADOS

El análisis estadístico evidenció diferencias significativas en la reducción de unidades formadoras de colonias (UFC /ml) entre los distintos tratamientos evaluados. El análisis de dos factores (tratamiento * luz) reveló una interacción significativa entre el tipo de tratamiento y la aplicación de luz (p < 0,001), lo que indica que la eficacia del tratamiento depende tanto de la presencia de luz como de la combinación específica de curcumina y EDTA empleada. Las comparaciones múltiples mostraron que los grupos tratados con curcumina, curcumina + 1% EDTA y curcumina + 17% EDTA, en presencia de luz, presentaron reducciones significativas de UFC /ml en comparación con sus respectivos controles sin luz y con los grupos tratados únicamente con EDTA. Entre ellos, los grupos curcumina + luz y curcumina + 1% EDTA + luz mostraron los valores más bajos de UFC /ml, y se destacaron como los más efectivos. Por otro lado, el análisis de un solo factor (tratamiento) indicó que todos los grupos difirieron significativamente (p < 0,001) del control positivo (clorhexidina), siendo esta última más efectiva que las combinaciones evaluadas de curcumina con EDTA (Tabla 1).

## DISCUSIÓN

Este estudio evaluó la actividad antibacteriana de la aPDT mediada por curcumina activada por luz LED azul, en combinación con EDTA en dos concentraciones (1% y 17%). Los resultados demostraron que la aplicación de la luz azul potenció la actividad antimicrobiana de la curcumina; sin embargo, la adición de EDTA no produjo una mejora significativa en dicha actividad. 

La curcumina ha sido ampliamente estudiada como fotosensibilizador en aPDT debido a su capacidad de activación por luz azul dentro del rango de 400-500 nm, con un pico máximo de absorción cercano a los 450 nm ^16^. Su eficacia antimicrobiana depende de múltiples variables, incluyendo la concentración utilizada, el tiempo de exposición y la intensidad de la fuente lumínica ^26^^,^^27^. En este sentido, la elección del dispositivo de luz azul se vuelve crucial para optimizar los resultados terapéuticos. 

Los hallazgos de este estudio confirman la eficacia antimicrobiana de la curcumina activada por luz LED azul. Sin embargo, la combinación con EDTA no incrementó significativamente el efecto antibacteriano, en contraste con estudios previos que sí reportaron mejoras al combinar EDTA con fotosensibilizadores ^23^^,^^27^. Esta discrepancia puede explicarse por diferencias metodológicas, tales como el tipo de fuente de luz empleada (mononda vs. polionda), la distancia de irradiación, el tiempo de exposición y el periodo de preirradiación. La eficiencia de activación de la curcumina depende críticamente de que la fuente lumínica emite longitudes de onda compatibles con su espectro de absorción, dentro de un intervalo temporal adecuado ^28^^,^^29^. 

Actualmente, en odontología se emplean principalmente dos tipos de unidades LED para fotopolimerización: las unidades mononda, que emiten en un rango estrecho (usualmente entre 450-470 nm), y las unidades polionda, que cubren un espectro más amplio (380-515 nm) ^30^. Algunas evidencias sugieren que las fuentes poliondas pueden ser más efectivas en contextos donde se requiere activación de diferentes fotosensibilizadores ^31^. Por ejemplo, Nima et al. (23) emplearon una unidad polionda (Bluephase G2) con resultados favorables, mientras que Méndez *et al*. ^32^ usaron una unidad mononda (Biotable) con menor eficacia. En el presente estudio se utilizó una unidad LED mononda con una longitud de onda de 480 nm, lo cual podría haber limitado la eficacia observada. Estudios como los de Dos Santos *et al*. y Terra-García *et al*. ^33^^,^^34^ respaldan esta observación, pues indican que las unidades LED mononda pueden presentar limitaciones en la activación eficiente de ciertos fotosensibilizadores, como la curcumina. 

Además del tipo de luz, el tiempo y la distancia de irradiación también son factores determinantes en la efectividad de la aPDT. Se ha reportado que tiempos de irradiación superiores a 60 segundos incrementan la actividad antimicrobiana de la aPDT con curcumina frente a *S. mutans* y *Candida albicans*^35^. Otro estudio mostró que una aplicación de 120 segundos tuvo como resultado una mayor reducción de *S. mutans* que exposiciones más breves ^36^. En nuestro protocolo, el tiempo de irradiación fue de 40 segundos, lo que posiblemente haya sido insuficiente para lograr una activación óptima del fotosensibilizador.

El tiempo de preirradiación, es decir, el intervalo entre la aplicación del fotosensibilizador y la exposición a la luz, también puede influir en los resultados. Fumes *et al*. ^37^ observaron que, aunque no hubo diferencias estadísticamente significativas entre diferentes tiempos de preirradiación (1, 2 y 5 minutos), períodos adecuados pueden optimizar la eficacia de la aPDT con láser de diodo. De igual manera, Furtado *et al*. ^38^ evaluaron tiempos de preirradiación entre 0 y 5 minutos utilizando azul de metileno y un láser de baja potencia, y con una distancia estandarizada para asegurar la absorción lumínica, sin encontrar diferencias significativas entre los tiempos evaluados. En este estudio, el tiempo de preirradiación fue de 40 segundos, lo cual podría no haber sido suficiente para favorecer una adecuada interacción entre la curcumina y la membrana bacteriana antes de la activación lumínica. Por lo tanto, tiempos mayores de preirradiación podrían mejorar la eficacia antibacteriana de la aPDT mediada por curcumina.

El EDTA es ampliamente utilizado en medicina y suele combinarse con antibióticos para potenciar su acción antimicrobiana. Altera la integridad de la membrana bacteriana; inhibe mecanismos de resistencia, como las bombas de eflujo, y favorece la penetración de compuestos activos, lo que lo consolida como un coadyuvante eficaz en terapias frente a cepas resistentes ^39^^,^^40^. En odontología, el EDTA al 17 % se emplea en endodoncia para remover el barrillo dentinario y facilitar la limpieza de los conductos radiculares ^25^, por lo que se evaluó esta concentración en busca de un mayor efecto. Sin embargo, nuestros resultados muestran que el incremento de la concentración no potenció el efecto antibacteriano. Otros factores, como el tiempo de preirradiación, deben investigarse para determinar si influyen en la eficacia antimicrobiana de la solución.

Algunas limitaciones de este estudio pueden haber afectado los resultados; entre ellas, el uso de un único tipo de fuente de luz LED (mononda) con una longitud de onda específica, lo cual pudo haber restringido la eficacia de activación de la curcumina. Asimismo, el tiempo de irradiación y de preirradiación fueron relativamente cortos en comparación con otros estudios, lo que podría haber influido en la actividad antimicrobiana observada. Además, el diseño *in vitro* no permite replicar completamente las condiciones clínicas, por lo que los resultados deben interpretarse con cautela al extrapolarlos al entorno clínico. 

Si bien diversos estudios han evaluado el efecto de la aPDT sobre *S. mutans* y otras especies bacterianas en distintos contextos, la evidencia específica sobre la aplicación combinada de PDT con curcumina y EDTA en la reducción de unidades formadoras de colonias (UFC) de *S. mutans* en restauraciones de resina compuesta sigue siendo limitada. Se requieren investigaciones adicionales para determinar con mayor precisión la eficacia, seguridad y aplicabilidad clínica de esta combinación terapéutica.

## CONCLUSIONES

La aPDT mediada por curcumina activada con luz LED azul mostró un efecto antimicrobiano contra *S. mutans*; sin embargo, ambas concentraciones del EDTA se comportaron de la misma manera, sin potenciar significativamente la efectividad antimicrobiana. Es necesario optimizar los parámetros de aplicación para mejorar su eficacia.

## Financiamiento:

Autofinanciado
